# Harnessing the Microbiome to Reduce Pancreatic Cancer Burden

**DOI:** 10.3390/cancers15092629

**Published:** 2023-05-05

**Authors:** Ana Raquel Bastos, Joana Pereira-Marques, Rui Manuel Ferreira, Ceu Figueiredo

**Affiliations:** 1 Instituto de Investigação e Inovação em Saúde, Universidade do Porto (i3S), 4200-135 Porto, Portugal; 2 Department of Pathology, Faculty of Medicine, University of Porto, 4200-319 Porto, Portugal; 3 Institute of Molecular Pathology and Immunology, University of Porto (IPATIMUP), 4200-135 Porto, Portugal

**Keywords:** microbiome, pancreatic cancer, pancreatic ductal adenocarcinoma, carcinogenesis, therapy response

## Abstract

**Simple Summary:**

Pancreatic cancer has one of the worst prognoses among all the malignancies. To date, there are no effective strategies for preventing, diagnosing, or treating this aggressive disease. Increasing evidence shows that the human microbiome influences pancreatic cancer development and the response to treatment, which is the main aim of this review. Future studies will be key to determining whether or not this knowledge might be used to develop new strategies that harness the microbiome for diagnostic and therapeutic interventions in pancreatic cancer.

**Abstract:**

Pancreatic cancer mortality is expected to rise in the next decades. This aggressive malignancy has a dismal prognosis due to late diagnosis and resistance to treatment. Increasing evidence indicates that host–microbiome interactions play an integral role in pancreatic cancer development, suggesting that harnessing the microbiome might offer promising opportunities for diagnostic and therapeutic interventions. Herein, we review the associations between pancreatic cancer and the intratumoral, gut and oral microbiomes. We also explore the mechanisms with which microbes influence cancer development and the response to treatment. We further discuss the potentials and limitations of using the microbiome as a target for therapeutic interventions, in order to improve pancreatic cancer patient outcomes.

## 1. Introduction

Pancreatic cancer ranks as the 12th most incident cancer and the seventh cause of cancer-related death worldwide, accounting for around 496,000 new cases and 466,000 deaths annually [1], with incidence and mortality being predicted to nearly duplicate in 2040 due to demographic changes [2].

Pancreatic cancer has a poor prognosis. The 5-year overall survival rate has increased by little with modern therapeutics, remaining as low as 5–10%, and remaining the worst among all types of cancers [3]. Pancreatic ductal adenocarcinoma (PDAC) accounts for more than 90% of pancreatic cancer diagnoses, arising from the ductal cells that constitute the exocrine portion of the pancreas. Many factors contribute to the overall bad prognosis of PDAC, including the absence of well-established risk factors and prevention strategies, the lack of a reliable screening tool, the aggressive biology of the tumor, the late diagnosis (generally made at the locally advanced or metastatic stage), and resistance to therapy.

Emerging evidence suggests that the microbiome influences pancreatic carcinogenesis, so identifying changes in the microbiome of patients with pancreatic cancer will likely set the stage for the development of novel screening, diagnostic, and therapeutic approaches for this deadly disease. In this review, we overview the current literature related to the microbiome and pancreatic cancer. A literature search was performed in the PubMed database, using a combination of terms, including “microbiome”, “microbiota”, “pancreatic cancer”, and “pancreatic ductal adenocarcinoma”, without restriction of the study design, publication date, or status. Studies not written in English were excluded. Additional studies were retrieved from the lists of references of selected articles. We aim to explore and summarize the latest findings on the mechanisms with which the microbiome modulates pancreatic cancer development and resistance to therapy. We further discuss potential therapeutic strategies to modulate the microbiome and briefly look into the challenges of future research on pancreatic cancer and the microbiome.

## 2. Pancreatic Cancer Risk Factors

PDAC is a paradigmatic example of an inflammation-driven cancer. Inflammation is both a risk factor for pancreatic cancer and also a consequence of carcinogenesis [4]. Oncogenic KRAS is the most common driver of mutation in human PDAC, being present in more than 90% of the cases [4]. There is a synergic relationship between KRAS activation and inflammation, as KRAS expression is itself highly proinflammatory, and the duration of pancreatitis is associated with the predisposition to KRAS mutations [5,6].

In addition to chronic pancreatitis, including hereditary and other forms, non-modifiable risk factors for PDAC include increasing age, familial cancer syndromes, being of the Afro-American race, and being of a non-O blood group [7]. Tobacco exposure and obesity are, so far, the only modifiable risk factors with convincing evidence to be considered well-established causes of pancreatic cancer [8]. A high-fat diet, diabetes [7], poor oral health status [9], and blood antibodies against selected oral pathogens [10,11] are also associated with an increased risk of pancreatic cancer. These risk factors are related with a state of chronic systemic inflammation that might be pro-tumorigenic, and are also associated with changes in the human microbiome that might be a common intermediate mechanistic step leading to pancreatic carcinogenesis [12].

## 3. The Human Microbiome

The human body is inhabited by a large collection of microorganisms that globally constitute the microbiota, and include bacteria, viruses, fungi, and protozoa [13]. The term microbiome generally refers to microorganisms, their genetic information, and the specific host environment which they inhabit, although currently the terms microbiota and microbiome are often used interchangeably [14]. The majority of the human microbiota reside in the gastrointestinal tract, skin and oral cavity, but organs previously considered to be sterile, such as the lung, the liver, and the pancreas, are now recognized as harboring low-biomass microbial populations. Whether microbiomes are natively established in these niches or represent the transient migration of microbes from adjacent sites is still unclear [13].

The composition of the normal microbiome varies significantly between individuals and between body sites, being influenced by local conditions, host genetics, diet, antibiotic consumption, and lifestyle [15]. Under healthy conditions, the commensal microorganisms symbiotically interact with the host across multiple body sites, playing an essential role in a variety of physiological processes. Disruption of the balance that exists between the microbiome and the host, called dysbiosis, may contribute to the pathogenesis of many diseases, including cancer [15].

## 4. The Microbiome in Pancreatic Cancer

### 4.1. The Intratumoral Microbiome

Cancer is classically considered a genetic disease, but tumors are much more than masses of abnormal proliferating cells [16]. Instead, the tumor microenvironment (TME) contains multiple distinct cell types that interact with one another in a complex way, contributing to the acquisition of hallmark traits that enable tumor growth and metastatic dissemination [16]. Since the microbiome is an intrinsic component of the tumor microenvironment, one may expect that the crosstalk between microbes and tumor cells is able to modulate oncogenesis.

Intratumoral bacteria were detected for the first time more than 100 years ago [17]. Currently there is strong evidence of an intratumoral bacterial microbiota across, at least, 33 major cancer types [18]. By directly analyzing the bacterial communities of tumors and their adjacent normal tissues in seven types of cancer, including the pancreas, Nejman et al. demonstrated that each tumor type has a distinct microbiota composition [19]. Consistently with these findings, predictive associations between different cancer types and specific microbiota profiles were recently reported by examining genome and transcriptome sequencing data of The Cancer Genome Atlas [18].

Several studies characterized the tumor microbiota in pancreatic cancer (Table 1).

Geller et al. compared pancreatic tumor tissues and normal pancreatic tissues and identified bacterial DNA in 76% of the tumors and in 15% of the normal tissues [24]. They identified Gammaproteobacteria as the most common taxa, including members of the Enterobacteriaceae and Pseudomonadaceae, with *Pseudomonas*, *Citrobacter*, *Klebsiella*, *Streptococcus*, and *Acinetobacter* as genera with the highest mean relative abundances. These data are consistent with those reported by Nejman et al. showing that more than 60% of pancreatic tumors were positive for bacterial DNA, predominantly belonging to the phyla Proteobacteria and Firmicutes, with a high prevalence of *Enterobacter asburiae, Klebsiella pneumoniae, Citrobacter freundii, and Fusobacterium nucleatum* [19]. These findings suggest a bacterial reflux from the duodenum to the pancreas via the major/minor papilla and the pancreatic duct, as Proteobacteria also dominate the normal duodenal microbiome [19,24]. Accordingly, it was also observed that bacterial DNA profiles were similar between the pancreas and duodenum of the same subjects, which further supports that bacteria migrate from the gut into the pancreas [25]. Nejman and colleagues also showed that the microbial populations present in the tumor tissues of different cancer types are similar to those of their normal adjacent tissues [19], leading to the hypothesis that the tumor microbiome evolves from microbes existing in normal tissues [26]. In contrast, Riquelme et al. found that the human gut microbiome represents approximately 25% of the PDAC microbiome, while it is absent in the normal adjacent tissue, suggesting that gut microbes specifically colonize pancreatic tumors [23]. Additional studies evaluating the intratumoral microbiota reported tumor enrichment in *Lactobacillus* spp., *Akkermansia muciniphila*, and *Bacteroides* [20], and *Pseudomonas*, *Herbaspirillum*, and *Sphingomonas* [21] in comparison with adjacent normal tissue.

It remains unknown how bacteria may colonize the pancreas, but studies with fluorescently labeled bacteria administered via oral gavage to mice revealed bacteria migration to the pancreas [27]. Inflammatory conditions associated with pancreatic cancer, such as pancreatitis and obesity, may induce gut dysbiosis and alter gut permeability, thus increasing microbial translocation to the pancreas [28].

As with the bacteriome, fungal dysbiosis has been identified in cancer and studies have linked fungi to pancreatic carcinogenesis [29]. Aykut et al. showed that PDAC tumors in both humans and mouse models display a 3000-fold increase in fungi compared to the normal pancreas, with higher mycobiome diversity and enrichment in *Malassezia* [30]. The authors demonstrated that fluorescently labeled fungi translocated from the gut lumen to the pancreatic tissue. Fungal ablation was protective against tumor development, while repopulation with *Malassezia*—but not *Candida*, *Aspergillus,* or *Saccharomyces*—accelerated oncogenesis. *Malassezia* was shown to promote pancreatic tumorigenesis via the activation of mannose-binding lectin and the subsequent activation of the complement cascade [30], which stimulates cell growth, survival and migration [31]. Fungal dysbiosis of the digestive tract is often characterized by an overgrowth of *Candida albicans*, which was independently associated with a higher PDAC risk in two large population-based cohorts in Sweden [32] and in Taiwan [33]. A valuable next step will be to determine whether or not the mycobiome interacts with the bacteriome to promote PDAC progression [31].

Clinical disease heterogeneity exists in pancreatic cancer, and since limited genomic differences have been found [34], the tumor microbiota has been studied as a possible factor that may determine disease prognosis. Pancreatic tumors located in the head of the pancreas are generally easier to detect, so their overall prognosis is better than that of pancreatic body/tail cancers. Recently, prognostic and genetic differences between pancreatic head and body/tail cancers were identified [35], but no differences in intratumoral bacteria diversity or relative abundance were detected between anatomical locations [36].

Long-term and short-term PDAC survivors have different intratumoral microbiota structures (Table 1), with higher bacterial diversity being associated with longer survival [22,23]. In two independent PDAC patient cohorts from the USA, Riquelme et al. identified an intratumoral microbial signature of *Pseudoxanthomonas*, *Streptomyces*, *Saccharopolyspora*, and *Bacillus clausii,* which was predictive of long-term survivorship [23]. Huang et al. identified an enrichment in *Sphingomonas*, *Megasphaera*, *Bradyrhizobium*, *Desulfovibrio*, *Flavobacterium*, *Enhydrobacter*, and *Megamonas* in long-term-surviving PDAC patients of Chinese origin [22]. Patients with high relative abundances of *Sphingomonas* and *Megasphaera* had longer overall survival, whereas patients with a high abundance of *Clostridium* had shorter survival. *Fusobacterium nucleatum,* an anaerobic Gram-negative species of the oral microbiome implicated in colorectal cancer, has also been identified as a prevalent species in pancreatic cancer [19] and associated with worse prognosis [37]. Notably, *Pseudomonas*, a common opportunistic pathogenic bacterium, was also observed to be enriched in PDAC patients with short-term survival [23] and was identified as the most abundant genus that translocates from the gut to the pancreas in PDAC patients [27].

Taken together, these data show that the composition of a tumor’s bacterial microbiota in pancreatic cancer is highly variable, although microbial signatures might be useful to predict pancreatic cancer prognosis. Once microbial biomarker signatures are identified both in pancreatic cysts or tumors, microbial phenotyping via endoscopic ultrasound might emerge as a new approach to screen, stratify the risk of and select therapeutics for pancreatic cancer [36]. Whether intratumoral bacteria play a causal role in pancreatic carcinogenesis or simply infect tumors already established is still not clear.

### 4.2. The Gut and the Oral Microbiomes in Pancreatic Cancer

Multiple studies in the last decade have explored the association of the human oral and gut microbiomes with pancreatic cancer, as these may represent non-invasive approaches to early cancer diagnosis (Table 2).

#### 4.2.1. The Gut Microbiome and Pancreatic Cancer

An expanding body of evidence has associated gut microbiome changes with pancreatic cancer. Studies investigating the link between the gut microbiome and pancreatic cancer have shown that the structure of the gut microbial communities of pancreatic cancer patients is clearly distinct from that of healthy controls, as evaluated by beta diversity analysis [38,41,42,43]. However, reports are inconsistent regarding the gut microbial alpha diversity in pancreatic cancer patients, with studies showing higher, similar, or lower diversity compared to that of healthy controls. Overall, microbial biomarker species have high accuracy to distinguish PDAC patients from healthy controls [20,38,39,41,42,43]. In the multinational study of Nagata and colleagues, dysbiosis of the gut microbiome was reported and significant associations were identified between PDAC and 30 gut bacterial species [38]. These bacterial signatures had high levels of accuracy in predicting PDAC in the Japanese discovery and in the Spanish and German independent patient cohorts. A common PDAC gut microbial signature in all these cohorts was the enrichment in *Streptococcus* and *Veillonella* spp. and depletion of *Faecalibacterium prausnitzii*.

Kartal et al. showed that fecal microbiome classifiers based on 27 bacterial species were better than those of the saliva microbiome in the identification of PDAC patients, in Spanish and German cohorts [20]. Others have shown an enrichment in pro-inflammatory genera, especially those belonging to the phylum Proteobacteria in the gut microbiome of PDAC patients [27,41]. In a Chinese cohort comparing the fecal microbiome of PDAC patients with that of matched healthy controls, the gut microbial community of PDAC patients had a higher abundance of lipopolysaccharide-producing bacteria, and a reduction in beneficial microbes, such as butyrate-producing bacteria [43]. Accordingly, significant reduction in Firmicutes, especially of butyrate-producing bacteria, was also observed in the gut microbiome of PDAC patients [41]. Taxa consistently identified as enriched in the gut microbiome of pancreatic cancer patients include *Veillonella* [20,27,38,40,41,42,43], *Streptococcus* [24,38,39,40], *Actinomyces* [22,38,40,47], *Lactobacillus* [20,39,40], and *Klebsiella* [27,39,43]. Taxa depleted in the gut microbiome of pancreatic cancer patients include *Faecalibacterium* [20,38,41,42], *Bifidobacterium* [20,39,43], *Eubacterium* [38,39,41], *Anaerostipes* [40,42,43], *Blautia* [39,43], *Coprococcus* [39,43], and *Clostridiales* [38,42]. Still, there is high microbial variability between individuals, both within and across different populations. The combination of fecal microbial patterns with additional biomarkers may be necessary to increase accuracy in pancreatic cancer detection, as shown by Kartal et al. who combined fecal microbiome classifiers with serum levels of CA 19-9 to improve accuracy in the identification of PDAC patients [20].

The association between the duodenal microbiome and pancreatic cancer has been scarcely explored, but it has been hypothesized that changes in the duodenal microenvironment might contribute to a higher incidence of pancreatic head cancer compared to pancreatic body/tail cancers [52]. The microbiome of different pancreatic sites appears to be similar to the duodenum’s microbiome of the same subjects [25]. Recently, it was shown that patients with PDAC have alterations in their duodenal fluid microbial profiles, showing higher levels of bacterial and fungal DNA, reduced microbial diversity and an enrichment in *Bifidobacterium* [53]. Additionally, duodenal fluid samples from short-term PDAC survivors were enriched with bacteria belonging to the genera *Fusobacterium*, *Rothia,* and *Neisseria*. Microbial profiling of duodenal fluid acquired via endoscopy to screen and stratify the risk of pancreatic cancer may deserve further attention. Factors that influence duodenal fluid composition, such as the use of proton-pump inhibitors, must be considered. The role of *Helicobacter pylori* in PDAC remains controversial [54], but it is possible that infection of the gastric body mucosa with subsequent hypochlorhydria may be associated with duodenal fluid alterations.

Compared to the bacterial populations in the human gut, the enteric mycobiome has low diversity (dominated by yeasts such as *Saccharomyces*, *Malassezia*, and *Candida*), with high intra-individual variability over time [55]. In contrast, viruses in the gut are highly diverse with limited intra-individual variability over time [56]. While some individual viruses have causal roles in carcinogenesis [57], the relationship between the gut virome and pancreatic cancer is far from being understood. A study in Japan reported differences in the gut virome structure between pancreatic cancer patients and healthy controls [39]. The gut virome of pancreatic cancer patients was enriched with *Cardiovirus*, *Alphabaculovirus,* and *Proboscivirus*, whereas 16 other viral genera were more common in the gut of healthy subjects [39]. The relative abundance of *Cyprinivirus* was positively correlated with the cancer stage [39]. The enteric virome consists largely of bacteriophages that infect specific bacterial species and help maintain a healthy gut microbiome [58]. Nagata et al. found 58 bacteriophages that potentially infect microbial species that are consistently enriched in the gut microbiome of patients with PDAC (*Streptococcus oralis, Streptococcus parasanguinis, Veillonella atypica,* and *Veillonella parvula*) [38]. Synergistic pathogenic interactions may exist, in which virome dysbiosis leads to the emergence and persistence of opportunistic bacteria that are able to modulate the development of cancer [29]. The characterization of the gut phageome will be fundamental before considering phage therapy as an approach to treat pancreatic cancer.

Despite the potential use of gut microbial signatures for cancer diagnosis, it remains unclear whether gut dysbiosis is a result or rather causally triggers the development of pancreatic cancer.

#### 4.2.2. The Oral Microbiome and Pancreatic Cancer

As for the gut microbiome, several reports have analyzed the link between the oral microbiome and pancreatic cancer (Table 2). The majority of studies, including those with a larger sample size, observed no differences in alpha diversity between cancer patients and controls [44,46,49,50], despite some reporting increased alpha diversity in the oral microbiota of pancreatic cancer patients [38,45]. Furthermore, while some studies show that the overall structure of the oral microbiome in pancreatic cancer patients can be distinguished from that of heathy control subjects [38,45,46], others do not identify such differences [48,49,50]. Still, there is supportive evidence that the oral microbiome plays a role in pancreatic cancer etiology, some of which stemmed from the hypothesis that the relationship between periodontal disease and pancreatic cancer is related to changes in the oral microbiota. A large study by Fan and colleagues, which analyzed oral wash samples from patients in the USA, identified two pathogens involved in chronic periodontitis, *Porphyromonas gingivalis* and *Aggregatibacter actinomycetemcomitans*, as being associated with increased risk of pancreatic cancer [48]. These results are in agreement with those of previous studies showing that patients with high levels of plasma antibodies against *P. gingivalis* were at higher risk of PDAC [10,11]. However, in a study of 273 pancreatic cancer cases and 285 controls from Iran, *Porphyromonas* was detected in similar proportions in cases and controls, and was not associated with pancreatic cancer [46]. Interestingly, in this study, the authors reported an increased abundance of *Haemophilus* being associated with decreased odds of pancreatic cancer, which corroborated previous findings in a population from the USA [49].

Farrell et al. identified various bacterial species that were significantly different between saliva samples of pancreatic cancer patients and healthy controls, including *Neisseria elongata* and *Streptococcus mitis* [51]. These two species, which were depleted in pancreatic cancer, were then validated in an independent cohort of patients and showed high sensitivity and specificity in distinguishing the two groups [51]. Other studies consistently report a decrease in the abundance of *Neisseria* in the oral microbiota of pancreatic cancer patients [45,47,49]. Depletion of various species of *Streptococcus* within a bacterial signature of 18 species was also identified in the oral microbiota of PDAC patients, which had high accuracy in predicting disease in Japanese, Spanish, and German patient cohorts [38].

In contrast to the above, other studies point to similar overall profiles in the oral microbiota of healthy controls and patients with pancreatic cancer, with no major differences in the abundance of taxa [20,44,49]. Whether oral microbiome signatures in pancreatic cancer patients represent risk factors for cancer development or are a result of the presence of cancer remains to be determined.

## 5. Mechanisms Linking the Microbiome to Pancreatic Cancer

The microbiome, both local and remote, can impact cancer development through a variety of mechanisms. These include the stimulation of oncogenic signaling pathways, the dampening of tumor suppressor mechanisms, the induction of DNA damage, the promotion of chronic inflammation, and the modulation of the tumor immune environment [59,60,61,62,63]. Microorganisms may influence carcinogenesis by binding to, invading, or injecting virulence factors into the host target cell, by secreting toxins and microbial metabolites, or by metabolizing host and dietary metabolites [13].

To evaluate the microbial metabolites affecting pancreatic carcinogenesis, Mendez et al. performed gut microbiome and metabolome analyses in a genetically engineered PDAC mouse model [64]. They showed that PDAC progression was accompanied by changes in the gut microbial population and, by characterizing the metabolome of the involved bacterial species, identified metabolites involved in polyamine metabolism [64]. The findings of elevated serum polyamine levels as tumors progressed in mice were also confirmed in human PDAC patients [64]. Other microbe-derived metabolites may have protective effects in cancer development, such as butyrate and pyridoxine [65]. Butyrate, a short-chain fatty acid (SCFA) produced by certain bacteria through the fermentation of non-digestible carbohydrates, was described to possess anti-inflammatory and anti-neoplastic properties [66]. Specifically, in the context of pancreatic cancer, butyrate was found to have “pro-differentiating, anti-proliferative, anti-invasive, pro-apoptotic” and chemo-sensitizing effects in PDAC cell lines in vitro [66]. Sodium butyrate also prevents the activation of pancreatic stellate cells, reducing the desmoplastic reaction in PDAC [67].

As mentioned above, a plausible explanation for the procarcinogenic effect of the microbiome involves chronic and low-grade activation of the immune system, which can perpetuate tumor-associated inflammation and immunosuppression [4,68]. It is likely that a persistent inflammatory state induced by microbial dysbiosis will result in excessive exposure to oxidative stress, leading to genetic instability and increasing the risk of tumorigenesis [69].

Since KRAS mutant signaling per se is not sufficient to initiate invasive PDAC, microbe-induced inflammation may potentiate pancreatic oncogenic signaling [70]. The local microbial activation of pattern recognition receptors, including toll-like receptor 4 (TLR4) [71], TLR7 [72], NOD-like receptor (NLRP3) inflammasome [73], and Dectin [74], may also potentiate mutant KRAS signaling and accelerate pancreatic oncogenesis. The activation of TLR2 and TLR5 was shown to suppress both innate and adaptive immunity in early and advanced PDAC [27]. Thus, the peri-pancreatic inflammatory response induced by the intratumoral microbiome may contribute not only to genetic oncogenic events, but also to helping shape an immunosuppressive microenvironment that promotes pancreatic cancer development [70]. Microorganisms remote from the pancreas, such as the gut and the oral microbiomes, may promote pancreatic carcinogenesis by triggering a systemic inflammatory response, or by reaching the pancreas (either through the bloodstream or the gastrointestinal tract) and locally modulate inflammation and the immune response [75].

Pancreatic cancer thrives in a profoundly immunosuppressive tumor microenvironment, which partially accounts for its aggressive biology. Tumors are characterized by a highly fibrotic stroma, often containing infiltration by pro-tumoral immune suppressor cells, including tumor-associated macrophages (TAMs), regulatory T cells (Tregs), and myeloid-derived suppressor cells (MDSCs) [29]. Cancer-associated fibroblasts (CAFs) and myofibroblast-like pancreatic stellate cells (PSCs) produce the collagen that composes the rich desmoplastic stroma of pancreatic tumors. TLR9 bacteria-induced stimulation may contribute to PSC activation [76]. Balachandran et al. demonstrated that tumors from long-term survivors of pancreatic cancer have a high quantity and diversity of neoantigens, which are associated with more abundant CD8+ T cell infiltrates [77]. Interestingly, the neoantigens exhibited homology to peptides derived from infectious agents, which is consistent with the existence of molecular mimicry between tumor neoantigens and microbial epitopes [77]. These data suggest that microbes might determine tumor immunogenicity and patient outcomes. Although PDAC tumors express neoantigens that can be recognized by T cells [78], there are adaptive immune suppression mechanisms that result in the inhibition of T cells’ cytotoxic activity, despite their presence in the tumor milieu [79].

Intratumoral bacteria are mainly found in the cytoplasm of tumor and immune cells [19]. They play a significant role in modulating relevant functions related to cancer hallmarks and immune activity, including cell motility, extracellular matrix interaction, complement cascade, and PD-1 signaling [19,80]. Interestingly, the tumor microbiome appears to have a dual effect, as it can promote either immune suppression or activation. Using genetically modified mouse models, Pushalkar et al. reported that bacteria induce intratumoral immune suppression mediated by TLR ligation, contributing to pancreatic cancer progression [27]. Bacterial ablation protected against PDAC progression and was associated with immunogenic reprogramming of the PDAC microenvironment, inducing an increase in intratumoral T cells and antitumoral M1 macrophages, along with a decrease in pro-tumoral MDSCs and M2 macrophages [27]. However, several studies suggested that the tumor microbiome might influence pancreatic cancer outcomes by activating the immune response. According to a recent study by Ghaddar et al., a high tumoral bacterial load was associated with decreased patient survival rates, high levels of tumor infiltration, T cell activation, and upregulation of PD-1-signaling pathways in both activated T cells and malignant cells [80]. Riquelme et al. also found that the tumor microbiome favors the recruitment and activation of CD8+ T cells into the tumor milleu, thus promoting the immune response against the tumor [23].

Overall, and in addition to identifying intratumoral populations of bacteria, fungi, and viruses, it is also important to understand the mechanisms through which these microorganisms interact with each other and with host cells in PDAC. Such knowledge could provide unique opportunities to improve clinical outcomes for patients with PDAC. For instance, understanding the role of viruses in the tumor microenvironment may enable the identification of phages that can selectively target and deplete tumor-promoting bacteria, instead of using non-selective antibiotics [68].

## 6. The Microbiome and Treatment Responses in Pancreatic Cancer

It is becoming increasingly evident that the microbiome has an impact on the efficacy of cancer treatment. However, the way the microbiome interacts with therapeutics is bidirectional and dynamic [14]. On the one hand, the microbiome can affect drug metabolism and absorption, modulating the efficacy and side effects of PDAC therapy [70], and on the other hand, drugs can shape the microbiome of the host.

### 6.1. Chemotherapy

Gemcitabine is a chemotherapeutic drug commonly used to treat PDAC. It has been shown that gemcitabine can be metabolized to its inactive form by the cytidine deaminase enzyme of Gammaproteobacteria, which are highly prevalent bacteria in human PDAC [24]. In a mouse model of colon carcinoma, gemcitabine-induced apoptosis was significantly enhanced when antibiotics were directly delivered into the tumor, suggesting a role of intratumoral bacteria in gemcitabine resistance [24]. However, gut dysbiosis induced by gemcitabine may paradoxically inhibit the drug’s anti-cancer effect and promote inflammation both locally and systemically. In a PDAC xenograft mouse model treated with gemcitabine, there was an overall increase in pro-inflammatory bacteria in the gut, with a reduction in *Lachnospiraceae* and *Ruminococcaceae*, two bacterial families known for their ability to generate butyrate [81]. Treatment with gemcitabine led to a significant reduction in serum levels of the anti-inflammatory compound inosine and its metabolites xanthine and hypoxanthine, which is likely associated with the observed dysbiosis [81].

Another chemotherapeutic option for treating advanced PDAC is the FOLFIRINOX regimen (5-FU, leucovorin, irinotecan, and oxaliplatin) [82]. Reactive oxygen species (ROS) are important for platinum-mediated cytotoxicity. The gut microbiome can increase oxaliplatin-induced ROS production, thus enhancing the efficacy of chemotherapy [83]. However, the gut microbiome may also increase the toxicity of chemotherapeutic drugs, thus limiting the effective dose that can be delivered [75]. An example is irinotecan-induced diarrhea promoted by an increase in β-glucuronidase-producing bacteria [84]. Further studies are needed to determine the impact of these drugs on the human gut microbiome and on PDAC prognosis in the clinical setting.

Neoadjuvant chemotherapy (NAC) is being employed more commonly in PDAC, not only to select and potentially increase the number of patients who can benefit from surgical resection, but also to reduce the chances of early recurrence and to improve peri-operative outcomes. Most patients receiving NAC undergo pre-operative biliary stenting, and accumulating evidence shows that these patients have a different bile microbiome compared to that of those undergoing primary surgery, with potential prognostic relevance [85]. Pre-operative biliary draining has been associated with higher risk of post-operative surgical site infections (SSIs), which is believed to be related to the introduction of intestinal bacteria into the biliary tree after stenting [85]. In a retrospective cohort, Nadeem et al. observed that NAC significantly alters the biliary microbiome of patients undergoing pancreaticoduodenectomy, as those undergoing NAC showed an increased growth of gram-negative anaerobes and an overall decrease in antibiotic resistance amongst biliary bacteria, particularly with the cephalosporin class of antibiotics [86]. These results are in contrast to those of another study in which resistance to cephalosporins was more common in patients who received NAC [85], which highlights the need to account for potential confounders in order to clarify the real impact of NAC in the biliary microbiome and thus tailor perioperative antibiotic prophylaxis. Postoperative pancreatic fistulas (POPFs) remain one of the most frequent and dangerous complications after pancreatic surgery, with common bacterial contamination resulting in higher morbidity and mortality [87]. While NAC appears to significantly reduce the incidence of POPFs [87], pre-operative biliary stenting may be an important risk factor for infectious complications after surgery [86]. Furthermore, a recent study demonstrated that the human bile reduces pancreatic cancer cell survival in vitro and peritoneal metastasis in vivo, with secondary bile acids showing more antitumor effects than primary bile acids do [88]. The anti-tumorigenic property of bile was reduced following pre-operative biliary stenting, presumably due to changes in the biliary microbiome [88].

### 6.2. Immunotherapy

Immunotherapy with immune checkpoint inhibitors, such as anti-PD-1, anti-PD-L1, and anti-CTLA-4, has shown remarkable success in several solid malignancies, but it has been ineffective for pancreatic cancer thus far, with the possible exception of very rare tumors with high microsatellite instability [89]. Commensal gut bacteria were found to positively influence the efficacy of PD-1-based immunotherapy in different types of epithelial tumors, with non-responders showing an imbalance in gut microbial composition that correlated with impaired immune cell activity [90]. *Bifidobacterium*, one of the major genera in the human gut microbiome, is believed to enhance the efficacy of the immune checkpoint blockers [91]. In several studies using mouse models of PDAC, it was observed that antibiotic-induced ablation of the gut microbiome may increase the sensitivity of the tumor to immune checkpoint inhibitors and reduce tumor burden [27,92,93]. Pushalkar et al. found that microbiome depletion promotes immunogenic reprogramming of the tumor microenvironment towards an activation phenotype, with increased PD-1 expression in intratumoral effector T cells [27]. Altogether, these studies suggest that antibiotic therapy combined with immune checkpoint blockers represents an attractive new strategy for treating patients with PDAC.

## 7. Microbiome Modulation in Pancreatic Cancer

Most patients with pancreatic cancer present with advanced unresectable disease, with treatment relying mainly on gemcitabine-based palliative chemotherapy. New therapeutic strategies have shown inconsistent results in PDAC, including neoantigen vaccines [94] and genomic targeted therapies [95]. Modulation of the host microbiome may represent a promising adjunct to current treatments for pancreatic cancer, as it may change the tumor microenvironment and sensitize tumors to therapeutics [96].

### 7.1. Diet

Changes in diet constitute an attractive approach to shape the microbial populations of the gut. A high-fat diet induces gut dysbiosis characterized by a reduction in the abundance of Bacteroidetes and an increase in the amount of Firmicutes and Proteobacteria, as well as gut inflammation and increased permeability, with the possible translocation of bacteria and its metabolites into the circulation [97]. Conversely, a high-fiber diet may protect against cancer, as it increases the microbial production of butyrate and other anti-inflammatory and pro-apoptotic SCFAs [98]. Engineered resistant starch (ERS) has emerged as a new strategy to enhance butyrate production and improve mucosal integrity [99]. Panebianco et al. found that an ERS diet retards tumor growth in a PDAC xenograft mouse model, by exerting a positive influence in the composition and metabolism of the gut microbiome [100]. Prospective clinical trials are necessary to determine how enriching a diet with nondigestible fibers may help to prevent and treat pancreatic cancer. Questions regarding the cost, acceptability, and potential adverse effects of these dietary interventions, such as gastrointestinal intolerance and enhanced cancer-associated cachexia, should be considered [13].

### 7.2. Probiotics

Probiotics are defined as “live microorganisms which when administered in adequate amounts confer a health benefit on the host” [101]. The range of microorganisms with potential health benefits is increasing in parallel to the accumulating knowledge on the human microbiome [96]. Probiotics have been suggested as an approach to cancer prevention and treatment, but in multiple cases have yielded conflicting results [13]. Gut dysbiosis in PDAC human patients is often characterized by a decrease in the abundance of butyrate-producing bacteria [41,43]. Regarding the aforementioned benefits of butyrate, accompanying conventional chemotherapeutic treatments with butyrate supplementation [66] and/or SCFAs-producing bacteria (e.g., *Faecalibacterium prausnitzii*) [102] may help to enhance clinical outcomes in pancreatic cancer. In addition, some probiotics can ameliorate the adverse effects of cancer therapies, such as chemotherapy-induced mucositis, thereby avoiding treatment discontinuation/dose reduction and increasing the chances of cure. In PDAC xenografted mice, the administration of probiotics enhanced the efficacy of gemcitabine while reducing chemotherapy-related toxicity [103]. As gemcitabine significantly alters the gut microbiome, and microbial changes may be detrimental for its efficacy, combined strategies using probiotics to restore a favorable gut microbiome may help in enhancing its effectiveness in the treatment of PDAC [81]. The administration of microorganisms associated with long-term cancer survival might also emerge as a new therapeutic strategy in PDAC [96]. Large clinical trials are required to develop precision probiotics that could address the heterogeneous microbiome and clinical features observed in different individuals [13].

### 7.3. Antibiotics

The use of antibiotics for microbial eradication in cancer is controversial, being currently recommended only for *H. pylori* erradication in gastric carcinoma and MALT lymphoma [13]. Preclinical studies have suggested a potential positive role of antibiotics in the treatment of PDAC, showing a reduction in tumor progression [27,92,93], and an improved response to anti-PD-1 immunotherapy [27] and to gemcitabine [24]. Antibiotic usage in metastatic PDAC was linked to increased survival in patients receiving gemcitabine-based chemotherapy, possibly due to a positive modulation of the microbiome, as well as a limitation of local and systemic infections [104]. Antifungal drugs were also found to slow PDAC progression and improve response to chemotherapy [30]. Long-term antibiotic treatments can have deleterious effects, such as drug toxicity, systemic microbial dysbiosis [14], the emergence of resistant strains, unforeseeable impacts on disease course and treatment efficacy [13], and possibly an increased risk of cancer [105]. In addition, by decreasing the abundance of commensal bacteria, broad-spectrum antibiotics can further exacerbate fungal colonization and infection [106]. Thus, strategies aiming to eliminate specific bacteria, rather than relatively untargeted approaches, might emerge as promising adjuncts to conventional cancer therapies [13]. One of such strategies may be phage therapy. Bacteriophages (or phages) are ubiquitous viruses that only infect bacteria, and have been recognized as promising tools to target specific disease-associated bacteria, while bearing minimal impacts on the surrounding microbiome. Alternatively, phages can be used as cargo particles to directly deliver drugs within the tumor microenvironment. Future research is needed to overcome the challenges that currently limit the widespread applicability of phage therapy [13,107].

### 7.4. Fecal Microbiota Transplantation

Fecal microbiota transplantation (FMT) refers to the transfer of the entire microbiota from a healthy individual into the intestine of a patient, aiming to restore the homeostasis of the gut microbiome [108]. In the last years, FMT has been investigated for the treatment of many intestinal and systemic disorders; notably, recurrent *Clostridium difficile* infections. Regarding cancer, FMT was shown to be a reasonable approach to mitigate the adverse effects of cancer treatment, such as colitis, and improve the efficacy of immune checkpoint inhibitors in melanoma [108]. Riquelme et al. found that the stools from PDAC patients with long-term survival harbor a specific bacterial signature that can alter the intratumoral microbiome and reduce tumor growth in fecally transplanted mice [23]. These findings suggest that FMT might be a way to modulate the microbiome in patients with PDAC, but future clinical trials are needed to validate these results. Importantly, FMT presents with several clinical, scientific, and regulatory uncertainties that need to be addressed before it can be routinely adopted in clinical practice [13]. For instance, effective donor and recipient selection, different regulatory policies across countries and institutions, and excessive costs may limit the widespread applicability of FMT. A future goal must be the identification of specific bacteria and microbial metabolites that drive the response to FMT, in the aim to develop personalized strategies to modulate the composition of the gut microbiome [13].

## 8. Concluding Remarks

**Figure 1:**
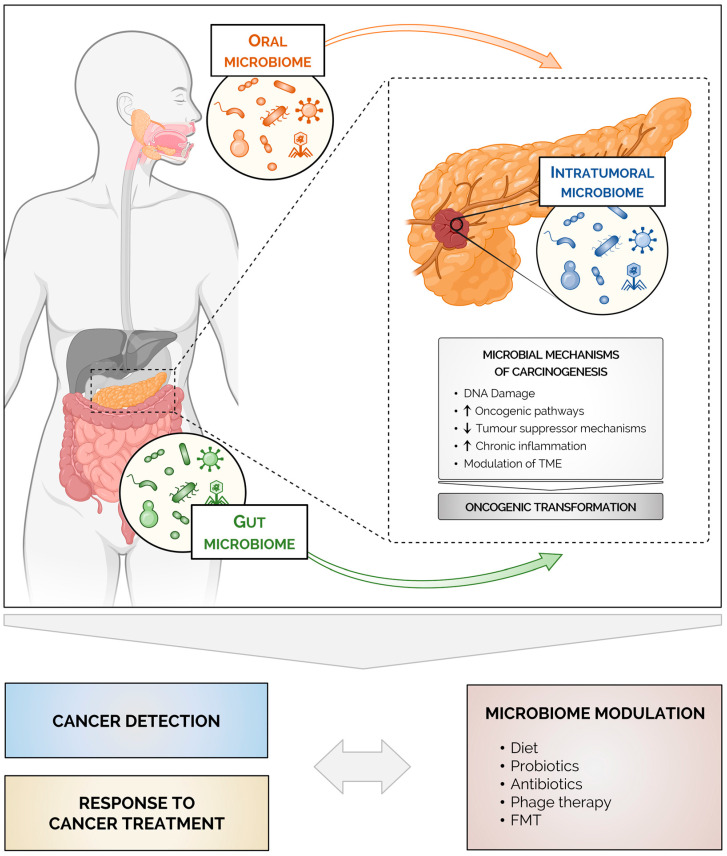
Microbes as an emerging tool for pancreatic cancer diagnosis and treatment. The microbiome influences pancreatic carcinogenesis both locally (intratumoral microbiome) and at a distance (gut and oral microbiomes). Mechanistically, microbially induced inflammation, metabolism and modulation of the tumor microenvironment contribute to the development of pancreatic cancer and to the overall poor response to chemotherapy and immunotherapy. Microbial signatures might be useful as non-invasive approaches for early detection and for defining the prognosis of pancreatic cancer. Ultimately, microbiome modulation may represent a promising adjunct to current treatments for pancreatic cancer. Created with BioRender.com.

The aggressive biology of pancreatic cancer remains poorly understood, but research on the microbiome has revealed that pancreatic cancer tissues harbor a distinct microbial community. Additionally, changes in the gut and oral microbiomes have been associated with pancreatic cancer progression and the response to treatment (Figure 1). However, consistent microbial signatures associated with pancreatic cancer features have not been disclosed. In addition to the low number of cancer patients included in some studies, factors such as the genetic background and geographic origins of the patients, as well as lifestyle habits and environmental exposure, may partially explain the differences observed between studies. Furthermore, differences in methods of sample collection, processing, analyzing, and microbiomic reporting may generate additional variation.

It needs to be highlighted that although bacteria are the most studied component of the microbiome in cancer, future research is needed to expand our understanding on how fungi and viruses interact with the bacterial microbiome and how they may influence pancreatic cancer development and treatment. Nevertheless, one of the major challenges in this area of research is to evolve from the identification of associations towards finding causality, since changes in the microbiome of pancreatic cancer patients may represent a risk factor for pancreatic cancer, but may also be the result of the overall cancer-induced disturbance in host homeostasis. Evidence from animal studies together with large prospective clinical studies will be fundamental to elucidating plausible mechanistic links and to definitely clarify whether microbial signatures are causative or the result of pancreatic cancer progression. Pancreatic cancer microbiome research will likely generate new knowledge, which may be translated into enhanced strategies for screening, diagnosis, and treatment of this deadly disease, eventually helping to reduce its burden.

## Figures and Tables

**Table 1:** Summary of studies evaluating the tumor microbiota in pancreatic cancer.

Reference	Country Disease (N)	Microbiota and Measurement	Major Findings
Kartal et al. 2022 [20]	Spain PDAC (50)	Tumor and adjacent normal tissue 16S rRNA gene sequencing	*Lactobacillus* spp., *Akkermansia muciniphila* and *Bacteroides* spp. enriched in tumor vs. adjacent normal tissue.
Tan et al. 2022 [21]	China PC (20)	Tumor and adjacent normal tissue 16S rRNA gene sequencing	No differences in alpha diversity and beta diversity between tumor and adjacent normal tissue; Forty-four genera were significantly different between PC and NAT; *Pseudomonas*, *Herbaspirillum* and *Sphingomonas* enriched in PC tissues.
Huang et al. 2022 [22]	China LTS-PDAC (17) STS-PDAC (13)	Tumor tissue 16S rRNA gene sequencing	No difference in Shannon index, but a higher number of species between LTS and STS; beta diversity analysis distinguished between LTS and STS; *Sphingomonas*, *Megasphaera*, *Bradyrhizobium*, *Desulfovibrio*, *Flavobacterium*, *Enhydrobacter*, and *Megamonas* were enriched in LTS, and *Clostridium*_sensu stricto 1, *Actinomyces*, *Porphyromonas*, *Aggregatibacter*, and *Neisseria* were enriched in STS.
Nejman et al. 2020 [19]	USA and Israel PC (67)	Tumor tissue 16S rRNA gene sequencing	Bacteria DNA in 68% of PC tumors; Proteobacteria dominated the microbiome of PC, followed by Firmicutes; *Enterobacter asburiae*, *Klebsiella pneumoniae*, *Citrobacter freundii*, and *Fusobacterium nucleatum* were the most prevalent species.
Riquelme et al. 2019 [23]	USA Discovery: LTS-PDAC (21) STS-PDAC (22) Validation: LTS-PDAC (36) STS-PDAC (32)	Tumor tissue 16S rRNA gene sequencing	Alpha diversity was higher in LTS vs. STS in both cohorts; Beta diversity distinguished LTS and STS in both cohorts; Patients with high microbial alpha diversity had significantly more prolonged OS than those with low alpha diversity did; *Saccharopolyspora*, *Pseudoxanthomonas*, and *Streptomyces* were significantly more abundant in LTS vs. STS, and were associated with significantly better outcomes.
Geller et al. 2017 [24]	USA and Israel NP (20) PDAC (113)	Tumor tissue and normal pancreatic tissue 16S rRNA gene qPCR and sequencing	Bacterial DNA in 76% of PDAC and in 15% of NP controls; the most common species were from Gammaproteobacteria, and most were members of the Enterobacteriaceae and Pseudomonadaceae, with *Pseudomonas*, *Citrobacter*, *Klebsiella*, *Streptococcus*, and *Acinetobacter* having highest mean relative abundances.

Alpha diversity is a quantitative measure of microbial diversity within samples; beta diversity is a measure of similarity between pairs of samples. LTS: long-term survivors; NAT: normal adjacent mucosa; NP: normal pancreas from organ donors; OS: overall survival; PC: pancreatic cancer; PDAC: pancreatic ductal adenocarcinoma; qPCR: quantitative real-time polymerase chain reaction; STS: short-term survivors.

**Table 2:** Summary of studies evaluating the gut and oral microbiota in pancreatic cancer.

Reference	Country Disease (N)	Microbiota and Measurement	Major Findings
Nagata et al. 2022 [38]	Japan, Spain, and Germany HC (235) PDAC (37)	Gut and oral Metagenomics	Alpha diversity decreased in the gut microbiome and increased in the oral microbiome of PDAC vs. HC; Significant differences in beta diversity for both the gut and oral microbiome between PDAC and HC; *Streptococcus oralis*, *Streptococcus vestibularis*, *Streptococcus anginosus*, *Veillonella atypica*, *Veillonella parvula* and *Actinomyces* sp. were enriched, and species of the order Clostridiales, such as *Lachnospiraceae*, *Eubacterium ventriosum*, and *Faecalibacterium prausnitzii* were depleted in the gut of PDAC; Unknown species of Firmicutes, *Dialister* sp., *Solobacterium* sp., *Prevotella pallens,* and *Prevotella* sp. C561 were enriched, and *Streptococcus salivarius*, *Streptococcus thermophilus*, and *Streptococcus australis* was depleted in saliva of PDAC.
Kartal et al. 2022 [20]	Discovery: Spain HC (50) CP (29) PDAC (57) Validation: Germany HC (32) PDAC (44)	Gut and oral Metagenomics	Fecal microbiome composition of PDAC different from that of HC and from that of CP; *Veillonella atypica*, *Fusobacterium nucleatum/hwasookii* and *Alloscardovia omnicolens* enriched and *Romboutsia timonensis*, *Faecalibacterium prausnitzii*, *Bacteroides coprocola* and *Bifidobacterium bifidum* were depleted in fecal microbiome of PDAC; No significant differences in taxa abundance in salivary microbiome. Fecal metagenomic classifiers were better than saliva-based classifiers in identifying PDAC patients in early- and late-disease stages; accuracy improved in combination with serum levels of CA 19-9.
Matsukawa et al. 2021 [39]	Japan HC (18) PC (24)	Gut and oral Metagenomics	Significant differences in gut bacterial and viral microbiomes between PC and HC; 26 species were significantly different between PC and HC, including *Klebsiella pneumoniae*, *Clostridium bolteae*, *C. symbiosum*, *Streptococcus mutans*, *Alistipes shahii*, *Bacteroides* spp., *Parabacteroides* spp., and *Lactobacillus* spp. being enriched, and *Collinsella aerofaciens*, *Blautia obeum*, *Bifidobacterium pseudocatenulatum*, *Eubacterium hallii*, *Lachnospiraceae*, *Coprococcus sp.*, and *Streptococcus sanguinis* being depleted in PC; Nineteen viral genera were significantly different in PC vs. HC.
Hashimoto et al. 2023 [40]	Japan HC (68) PDAC-bt (5)	Gut 16S rRNA gene sequencing	No differences in alpha diversity and beta diversity between PDAC-bt and HC; *Actinomyces*, *Streptococcus*, *Veillonella*, and *Lactobacillus* were enriched, and Anaerostipes was depleted in the fecal microbiome of PDAC-bt patients.
Zhou et al. 2021 [41]	China HC (32) PDAC (32)	Gut Metagenomics	Beta diversity analysis distinguished PDAC and HC; Proteobacteria (Gammaproteobacteria) and Fusobacteria were enriched and Firmicutes was depleted in PDAC; 24 species were differentially abundant in PDAC vs. HC, including *Escherichia coli*, *Fusobacterium nucleatum*, *Clostridium* spp., *Megamonas* spp., *Veillonella atypica*, *Veillonella parvula,* and *Prevotella stercorea*, which were enriched in PDAC, and *Faecalibacterium prausnitzii*, *Eubacterium rectale*, *Roseburia intestinalis*, and Ruminococcus sp., which were depleted in PDAC.
Half et al. 2019 [42]	Israel HC (13) PCL (6) PC (30) NAFLD (16)	Gut 16S rRNA gene sequencing	No differences in alpha diversity between HC, PCL and PC; beta diversity analysis distinguished PC and HC; *Bacteroidales*, *Odoribacter*, *Lachnospiraceae*, *Veillonellaceae*, *Megasphaera*, and *Akkermansia* were enriched, and *Clostridiales*, *Clostridium*, *Anaerostipes*, *Ruminococcaceae*, *Faecalibacterium*, and *Subdoligranulum* were depleted in PC.
Pushalkar et al. 2018 [27]	USA HC (31) PDAC (32)	Gut 16S rRNA gene sequencing	Alpha diversity was higher in PDAC than in HC; Genera of Firmicutes, Proteobacteria, Actinobacteria, and Bacteroidetes, including *Parabacteroides*, *Veillonella*, *Klebsiella*, and *Cardiobacterium* were enriched and *Megamonas* and *Prevotella* were depleted in PDAC.
Ren et al. 2017 [43]	China HC (57) PC (85)	Gut 16S rRNA gene sequencing	Alpha diversity was lower in PC vs. HC; Beta diversity analysis distinguished PC and HC; 15 taxa including *Prevotella*, *Veillonella*, *Klebsiella*, *Selenomonas*, and *Enterobacter* were enriched, and 25 taxa including *Bifidobacterium*, *Coprococcus*, *Clostridium* IV, *Blautia*, *Flavonifractor*, and *Anaerostipes* were depleted in PC.
Petrick et al. 2022 [44]	USA (African-Americans) HC (354) PC (122)	Oral Metagenomics	No differences in alpha diversity and beta diversity between PC and HC; No individual microbial taxa were differentially abundant between PC and HC after multiple comparisons.
Sun et al. 2020 [45]	China HC (10) BPD (17) PC (10)	Oral 16S rRNA gene sequencing	Alpha diversity was higher in BPD and PC vs. HC; Beta diversity distinguished PC, BPD and HC; *Fusobacterium*, *Megasphaera*, *Prevotella*, *Spirochaeta*, and *Treponema* were enriched, and *Leptotrichia* and *Neisseria* were depleted in PC; *Selenomonas* was enriched in BPD.
Vogtmann et al. 2020 [46]	Iran HC (285) PDAC (273)	Oral 16S rRNA gene sequencing	No differences in alpha diversity between PDAC and HC; Beta diversity analysis distinguished PDAC and HC; *Haemophilus* abundance was associated with increased risk of PDAC; Presence of *Enterobactericeae* and *Lachnospiraceae* was associated with increased risk of PDAC.
Wei et al. 2020 [47]	China HC (69) PDAC (41)	Oral 16S rRNA gene sequencing	Higher number of species and species richness and lower microbial diversity in PDAC vs. HC; *Streptococcus*, *Actinomyces*, *Rothia, Leptotrichia*, *Lactobacillus*, *Escherichia coli*, and Enterobacteriales were enriched, and *Selenomonas*, *Porphyromnas*, *Prevotella*, *Capnocytophaga*, *Alloprevotella*, *Tannerella*, and *Neisseria* were depleted in PDAC.
Fan et al. 2018 [48]	USA HC (371) PC (361)	Oral 16S rRNA gene sequencing	No differences in beta diversity; *Porphyromonas gingivalis* and *Aggregatibacter actinomycetemcomitans* were associated with higher PC risk, and Fusobacteria and *Leptotrichia* were associated with lower PC risk.
Olson et al. 2017 [49]	USA HC (58) IPMN (39) PDAC (34)	Oral 16S rRNA gene sequencing	No differences in alpha and beta diversity between HC, IPMN and PDAC; Firmicutes was enriched in PDAC, and *Haemophilus* and *Neisseria* enriched in IPMN and HC.
Torres et al. 2015 [50]	USA HC (22) OD (78) PC (8)	Oral 16S rRNA gene sequencing; qPCR *Leptotrichia*	No differences in alpha and beta-diversity between HC, OD, and PC; Significantly higher ratio of *Leptotrichia* to *Porphyromonas* in PC vs. HC or OD.
Farrell et al. 2012 [51]	USA Discovery: HC (10) PC (10) Validation: HC (28) PC (28)	Oral Discovery: Oligonucleotide HOMIM array Validation: 16S rRNA gene qPCR	Discovery: 16 species were significantly different between PC and HC, and 6 were validated by qPCR (*Atopobium parvulum*, *Granulicatella adiacens*, *Neisseria elongata*, *Prevotella nigrescens*, and *Streptococcus australis*, and *Streptococcus mitis*) Validation: *N. elongata* and *S. mitis* decreased in PC vs. HC; *G. adiacens* increased in PC vs. all non-cancer subjects.

BPD: benign pancreatic disease; CP: chronic pancreatitis; HC: healthy controls; IPMN: intraductal papillary mucinous neoplasms; NAFLD: non-alcoholic fatty liver disease; OD: other disease; PC: pancreatic cancer; PCL: pre-cancerous lesions; PDAC: pancreatic ductal adenocarcinoma; PDAC-bt: PDAC located in the body or tail of the pancreas; qPCR: quantitative real-time polymerase chain reaction.
